# Reshaping youth mental health care for optimal system-level outcomes: A dynamic modelling analysis

**DOI:** 10.1371/journal.pmen.0000232

**Published:** 2025-02-24

**Authors:** Adam Skinner, Eloisa Perez-Bennetts, Frank Iorfino, Mathew Varidel, Matthew Richards, Jo-An Occhipinti, Yun Ju Christine Song, Sebastian Rosenberg, Elizabeth M. Scott, Ian B. Hickie

**Affiliations:** 1 Brain and Mind Centre, Faculty of Medicine and Health, University of Sydney, Sydney, Australia,; 2 Computer Simulation and Advanced Research Technologies (CSART), Sydney, Australia; PLOS: Public Library of Science, UNITED KINGDOM OF GREAT BRITAIN AND NORTHERN IRELAND

## Abstract

Despite the critical role of effective treatment in limiting the adverse socioeconomic and health impacts of youth mental disorders, investment in mental health services is widely recognised to be insufficient to meet current demand for care. Using a dynamic model of resource-constrained services provision (constant total expenditure), we assessed the potential for improving mental health care outcomes for young people (aged 12–25 years) through changes in services system composition (i.e., the division of total services capacity between primary care services and specialised services) and policies for managing initial access to specialised treatment. Model analysis indicates that services delivery reforms facilitating direct engagement with specialised care, irrespective of clinical stage, are capable of significantly improving system-level (aggregate) outcomes, reducing total illness progression and disengagement from services, and increasing total treatment-mediated recovery. Restricting the ability of young people with relatively mild mental health problems to access specialised care (an explicit aim of stepped care models) effectively constrains the capacity of specialised services to promote recovery and prevent illness progression (both of which are more probable at earlier clinical stages), and does not make best use of available resources; better system-level outcomes are achieved when young people receive more effective, specialised treatment as early as possible. Provided that young people are not restricted to accessing specialised care only via primary care services, progression and disengagement decline as the proportion of mental health care expenditure allocated to specialised services increases, while the optimal proportion of young people directly accessing specialised care approaches a value of 1, suggesting that the largest improvements in services system performance are likely to be achieved by expanding specialised services capacity with the aim of providing all young people presenting for care with direct access to specialised treatment.

## Introduction

Mental health problems are the principal cause of disability in young people globally. Among adolescents and young adults aged 10–24 years, mental disorders were responsible for 28.2 million years lived with disability (YLDs) in 2019, nearly double the number of YLDs for the second-ranked Level 2 cause (neurological disorders, 14.7 million YLDs) [1]. Effective mental health care is critical not only for directly reducing this substantial disease burden (i.e., via treatment-mediated recovery), but also for preventing longer-term adverse socioeconomic and health outcomes that are associated with the development of serious mental health problems in adolescence and early adulthood, including economic non-participation, unemployment, welfare dependency, low educational attainment, and poor physical health [2–4]. Psychological and educational interventions are at least moderately effective in restricting disease progression in young people experiencing less severe anxiety and depressive symptoms [5–7], while evidence from prospective cohort studies indicates that relatively mild episodes of mental illness during adolescence (as opposed to more severe, prolonged and/or recurrent episodes) do not significantly increase the risk of psychopathology and associated deficits in social and occupational functioning in adulthood [8,9]. Access to mental health services therefore has the potential to prevent persistent (often lifelong) social and economic disadvantage, while also reducing the substantial burden of current symptoms [10].

Despite the critical role of effective treatment in limiting the adverse socioeconomic and health impacts of youth mental disorders, investment in specialised mental health services is widely recognised to be insufficient to meet current demand for care, particularly (although not only) in low- and middle-income countries [11,12]. Stepped care models, in which patients receive intensive, specialised mental health care only after failing to respond to less-intensive evidence-based treatments (typically provided in primary care), have been adopted in several countries as a means of managing severe constraints on specialised services capacity, since they ensure that patients are referred to specialised services only where this is necessary and may substantially reduce the need for specialised services to conduct comprehensive initial assessments of new patients as they engage with the health care system [13–15]. Nevertheless, stepped care approaches have also been criticised for deliberately and unnecessarily delaying access to appropriate treatment (i.e., for patients who require specialised services), prolonging distress and increasing the risk of progression to more severe disease [15,16]. Alternative approaches to mental health services delivery in which the intensity of care patients initially receive is based on symptom severity and level of functioning at the time of presentation (stratified care) are in principle capable of minimising delays in the provision of effective clinical interventions [15]; however, the potential benefits of these approaches will generally depend on the availability of specialised services, so that in practice, their ability to improve patient and system-level outcomes may often be limited (assuming more severely ill patients receive specialised care preferentially under a stratified care model, specialised services operating at maximum capacity will treat a similar number and mix of patients each year under stepped care and stratified care).

Here, we use a dynamic model of primary and specialised mental health services provision to examine the potential for improving mental health care outcomes for young people (aged 12–25 years) via the optimisation of services system composition and initial access to specialised care. Our analyses address two principal questions that have significant implications for policy decisions aimed at maximising services system effectiveness and efficiency: 1) what proportions of mental health care expenditure should be allocated to specialised services (psychiatry, clinical psychology, multidisciplinary mental health care, etc.) and primary care services (including general practitioner and youth-specific services) [15,17], assuming total expenditure is fixed and that specialised care costs more per patient per year than primary care (on average)? and 2) how should access to specialised services be managed for young people entering the services system (specifically, should a stepped care model, stratified care, or some other approach be adopted)? Additionally, we assess the degree to which embedding readily scalable e-health interventions (self-directed online care) [18] in routine clinical practice can help to reduce total rates of illness progression and disengagement from treatment and increase the total rate of recovery (our primary measures of services system performance). An accessible, public health-focussed introduction to the dynamic modelling methods used in our analyses is provided in ref. [19].

## Methods

### Model structure and assumptions

**Fig 1:**
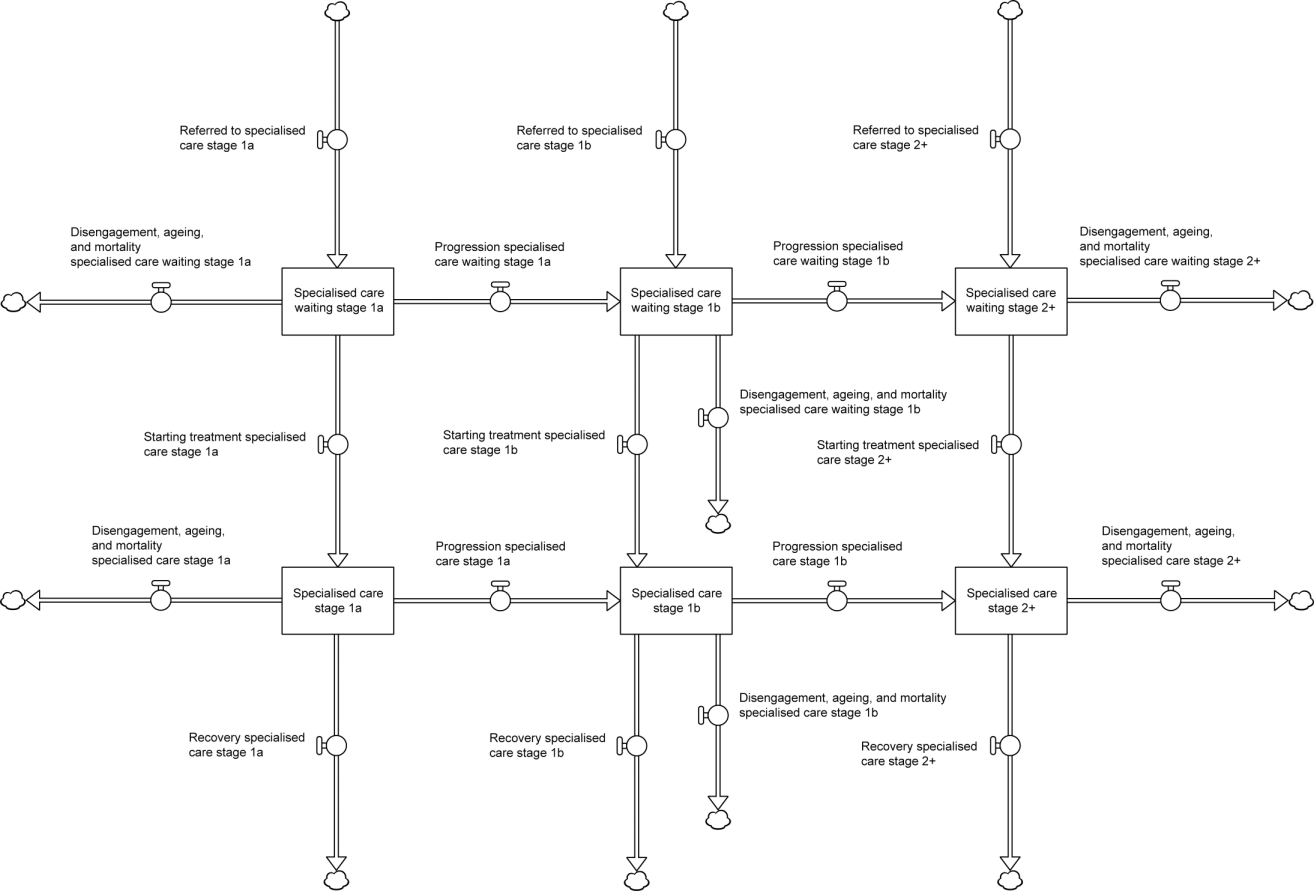
Structure of the specialised mental health services component of the model. Detailed descriptions of all model components are provided in S1 Appendix.

Fig 1 presents the structure of the specialised services component of the model, which consists of six stocks, or compartments (represented using boxes) [19], corresponding to numbers of adolescents and young adults (young people, aged 12–25 years) with relatively mild symptoms (clinical stage 1a) [20,21], more severe sub-threshold symptoms (clinical stage 1b), and full-threshold mental disorders (clinical stages 2–4) who are waiting for or receiving specialised mental health treatment. Adolescents and young adults referred to specialised services (either directly or via primary care services) flow into the waiting stocks in the upper half of Fig 1 (labelled ‘Specialised care waiting stage 1a’, etc.), where they remain until they commence treatment or leave the services system due to disengagement, ageing (i.e., reaching 26 years of age), or mortality. Treatment initiation rates are equal to $p_{i}\left(C-\underset{i}{\sum }T_{i}\right)/d$ , where $p_{i}$ is the proportion of young people waiting for specialised treatment at clinical stage *i* (1a, 1b, or 2–4), *C* is specialised services capacity (the maximum possible number of patients receiving specialised care at any point in time), $T_{i}$ is the number of young people at clinical stage *i* currently receiving specialised care, and the parameter *d* determines how rapidly new patients start treatment after existing patients are removed from the specialised care stocks through recovery, disengagement, mortality, and ageing (*d* was set to 1 week for all analyses). Progression from stage 1a to stage 1b and from stage 1b to stages 2–4 is assumed to occur at constant per capita rates, estimated using data for young people attending early intervention mental health services in Australia (see Table 1).

**Table 1:** Model parameter estimates. Distributions are given for parameters included in the sensitivity analyses (see Methods section).

Parameter	Value	Notes
Mental health care engagement rate per year	100000	Number of adolescents and young adults engaging with mental health services per year
Proportions of adolescents and young adults engaging with care at each clinical stage	0.467 (stage 1a); 0.238 (stage 1b); 0.296 (stage 2+)	Derived from national headspace data presented in [21]
Per capita rate of progression from stage 1a to stage 1b per year	Mean 0.253, 95% equal-tail interval 0.213–0.294 (lognormal distribution)	Derived from estimates reported in [22]
Per capita rate of progression from stage 1b to stage 2+ per year	Mean 0.060, 95% equal-tail interval 0.047–0.073 (lognormal distribution)	Derived from estimates reported in [22]
Per capita disengagement rate per year	0.262	Derived from [23]
Per capita mortality rate per year, stages 1a and 1b	0.000213	Derived from data published by the Australian Bureau of Statistics [24]
Mortality hazard ratio, stage 2+	2.22	Derived from [25]
Total services capacity	151703–187210	Maximum number of patients receiving care at any point in time, specified such that waiting times for primary care services and specialised services align with empirical estimates for Australia (16.3 days for primary care services, 41 days for specialised services) [26,27]
Specialised services proportion of total services capacity (scenarios a–d, Fig 5)	Mean 0.699, minimum 0.613, maximum 0.785 (uniform distribution)	Derived from national data on mental health services provision published online by the Australian Institute of Health and Welfare (available at: https://www.aihw.gov.au/mental-health/resources/data-tables)
Specialised services cost ratio (scenarios e–f, Fig 5)	1.44	Derived from national data on mental health care expenditure published online by the Australian Institute of Health and Welfare (Available at: https://www.aihw.gov.au/mental-health/resources/data-tables)
Per capita recovery rate per year, primary care	0.259 (stage 1a); 0.129 (stage 1b); 0.091 (stage 2+)	Derived from [20]
Recovery rate ratio, specialised care	Mean 1.921, 95% equal-tail interval 1.490–2.477 (lognormal distribution)	Derived from [28]
Recovery rate ratio, self-directed care (e-health interventions)	1.289	Pooled odds ratio reported in [18]

The primary care and self-directed (e-health) services components of the model are structurally identical to the specialised services component, except that: 1) there are outflows from the primary care treatment stocks capturing referrals to specialised services (i.e., as well as the outflows for recovery, disengagement, ageing, and mortality); and 2) there are no waiting stocks for self-directed online care (young people flow directly into the treatment stocks), since services capacity is effectively unconstrained (all model components are described in detail in S1 Appendix). Per capita recovery rates are assumed to be higher for patients receiving specialised care than for those receiving primary care (see Table 1) and are lower for patients with more severe symptoms (i.e., those at later clinical stages; Table 1). A recent meta-analysis of data from nine randomised controlled trials indicated that e-health interventions for depression in primary care are more effective than treatment-as-usual (pooled odds ratio 1.29), although significant differences between e-health interventions and usual care were reported in only a third of trials [18]. For analyses examining the effect of embedding e-health interventions (self-directed online care) in routine clinical practice, we ran two sets of simulations, one in which per capita recovery rates for each clinical stage were assumed to be the same for primary care and self-directed online care, and another in which per capita recovery rates for self-directed online care were assumed to be 1.29 times the per capita recovery rates for primary care.

### Model analysis

Services system performance under alternative policy scenarios (different levels of expenditure on specialised services, alternative approaches to managing initial access to specialised care, different rates of referral to self-directed online care) was assessed based on comparisons of equilibrium rates of illness progression, disengagement, and recovery, computed via simulation assuming 10^5^ adolescents and young adults flow into the services system every year. Simulations were run for a sufficient period (10^3^ model years) that the values of all stocks stabilised, at which point, the number of young people leaving the services system per year due to disengagement, recovery, ageing, and mortality is equal to the constant engagement rate (10^5^ people per year); we then used the stable stock values to calculate numbers of young people recovering, disengaging, and progressing from stage 1a to stage 1b and from stage 1b to stages 2–4 per year. The effect of the proportion of total mental health care expenditure allocated to specialised services on services system performance was examined assuming patients are referred to specialised care only via primary care services and assuming optimal stage-specific referral of new patients directly to specialised care; i.e., assuming the proportions of young people at each clinical stage referred directly to specialised care are such that total illness progression and disengagement per year (at equilibrium) are minimised. Optimal rates of direct referral to specialised services were calculated via constrained optimisation, implemented in Stella Architect version 3.3.0 (see www.iseesystems.com), assuming no referrals from primary care services to specialised services (optimisation analyses used the differential evolution algorithm, with the number of generations set to 40, a population size of 200, and default values for the remaining options). Constrained optimisation analysis was also used to determine optimal stage-specific rates of direct referral to self-directed online care for young people not referred directly to specialised services.

### Parameter values and sensitivity analyses

Model parameter values are presented in Table 1. Estimates of the proportions of young people at each clinical stage at the time of initial presentation to mental health services (assumed to be constant) were derived from national data on 12–25-year-olds attending headspace services in Australia over the period 2013 to 2017 [21]. For analyses in which patients are referred to specialised services only via primary care services (consistent with a stepped care approach), we specified per capita rates of referral from primary care services to specialised care for patients at each clinical stage such that waiting times for primary care services and specialised services at equilibrium were equal to empirical estimates for Australia (16.3 days for primary care services, 41 days for specialised services) [26,27]. Sensitivity analyses were performed to assess the impact on the simulation results of uncertainty in estimates of selected parameters, including the per capita rates of progression from stage 1a to stage 1b and from stage 1b to stages 2–4 per year, specialised services capacity (as a proportion of total services capacity, where expenditure on specialised services was not optimised as part of the analysis), and the recovery rate ratio for specialised care (i.e., the per capita recovery rate for patients at a given clinical stage receiving specialised treatment divided by the corresponding per capita rate for patients receiving primary care). Latin hypercube sampling was used to draw 100 parameter vectors, each containing a single value for each parameter, from a relatively broad (joint) distribution of values (see Table 1), and then for each parameter vector we computed equilibrium rates of disease progression, disengagement, and recovery for all relevant policy scenarios (as described in the previous section).

## Results

**Fig 2:**
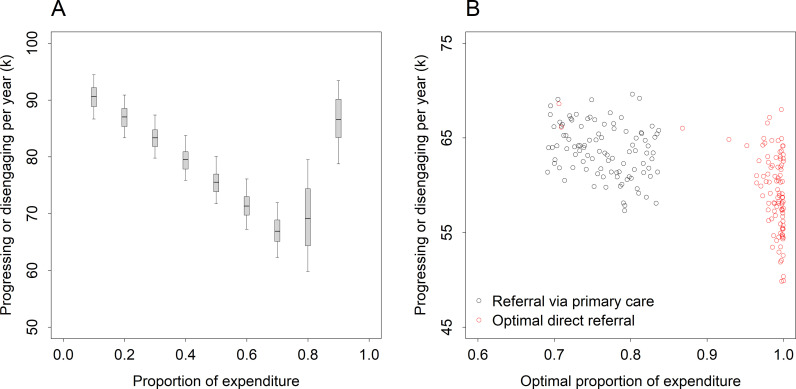
Effect of the proportion of total expenditure allocated to specialised services on illness progression and disengagement. Panel A—numbers of patients progressing or disengaging per year as a function of expenditure on specialised services, assuming patients are referred to specialised services only via primary care services (stepped care). Panel **B—**optimal expenditure on specialised services (i.e., the proportion of expenditure allocated to specialised services that minimises the total illness progression and disengagement rate) assuming patients are referred to specialised services only via primary care services (black points; scenario e in Fig 5), and assuming optimal direct referral to specialised services (red points; scenario f in Fig 5), i.e., assuming the proportions of patients at each clinical stage referred directly to specialised care are such that total illness progression and disengagement per year are minimised. Mean values (horizontal bars), 50% and 95% intervals (boxes and error bars, respectively), and individual points presented in all Figs are derived from the sensitivity analyses (see Methods section; Table 1).

Panel A in Fig 2 shows the effect on illness progression and disengagement (i.e., at equilibrium) of varying the proportion of mental health care expenditure allocated to specialised services, assuming adolescents and young adults are referred to specialised services only via primary care services (stepped care). Specialised care is assumed to cost more per patient per year than primary care (cost ratio 1.44; see Table 1), so that total services capacity decreases as the proportion of expenditure allocated to specialised services increases (total expenditure is constant); however, because specialised care is assumed to be more effective than primary care (mean per capita recovery rate ratio 1.92; Table 1), increased investment in specialised services also reduces the average length of time young people spend in treatment before recovering. The simulation results in panel A of Fig 2 indicate that total illness progression and disengagement per year declines as the proportion of expenditure allocated to specialised services is increased from 10% up to *c*. 70%. Past this point (i.e., for allocations greater than 70%), the negative effect of increasing waiting times associated with decreasing primary care services capacity more than offsets the positive effect of more rapid recovery associated with increasing specialised services capacity, and total illness progression and disengagement increases. Allowing young people to access specialised services directly effectively removes the trade-off between primary care waiting times and time to recovery, since patients are no longer required to flow through the primary care waiting stocks before receiving specialised treatment; in this case, the benefit of more rapid recovery (due to increasing access to specialised care) outweighs the cost of decreasing total services capacity, so that illness progression and disengagement are minimised when all (or nearly all) mental health care expenditure is allocated to specialised services (panel B in Fig 2, red points).

**Fig 3:**
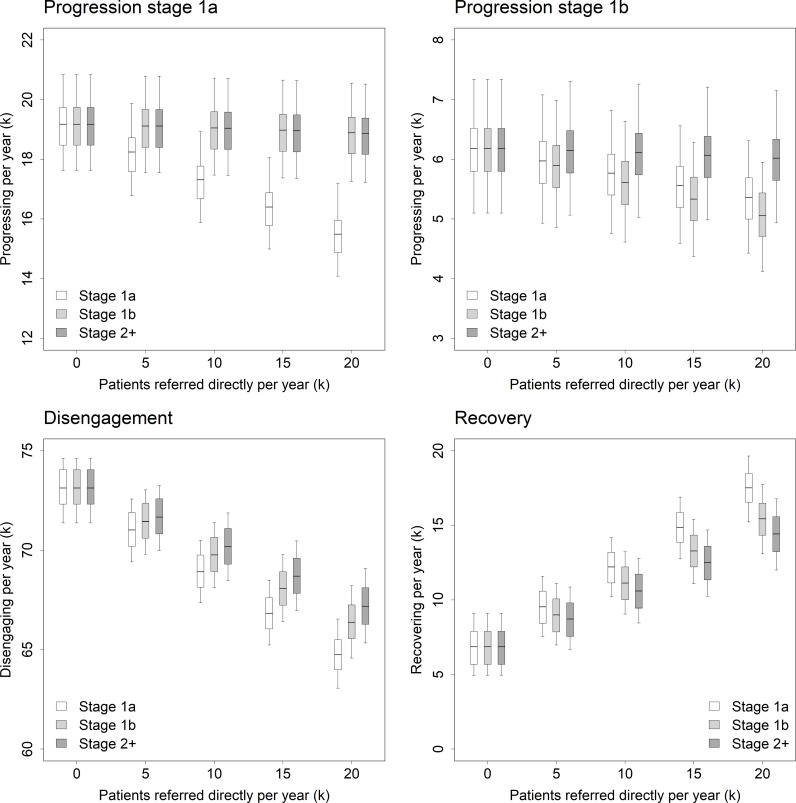
Effects of direct referral to specialised care on illness progression, disengagement, and recovery. Each panel shows the stage-specific effects of directly referring an increasing number of patients to specialised services on the total number of patients progressing, disengaging, or recovering per year (the lower right plot, for example, enables us to compare the effect on recovery of directly referring 10^4^ patients at stage 1a to specialised services per year with the effect of directly referring 10^4^ patients at stage 1b to specialised services per year). Per capita rates of referral from primary care services to specialised services are set to zero in all cases.

Direct referral of adolescents and young adults at stage 1a to specialised services is more effective in improving system-level outcomes than direct referral of the same number of young people at stage 1b, producing lower rates of illness progression and disengagement and a higher recovery rate (i.e., at equilibrium; see Fig 3). Similarly, improvements in system-level outcomes observed when young people at stage 1b are referred directly to specialised services are consistently greater than those observed when the same number of young people at stages 2–4 are referred directly to specialised care. The reason for this counter-intuitive (or at least non-obvious) result is that young people at earlier clinical stages recover more rapidly with treatment and are more likely to progress to later stages while waiting for care (young people at stages 2–4 necessarily have an illness progression rate of zero in our model). Providing specialised treatment to a young person at stage 1a is therefore more likely to promote recovery and prevent illness progression than providing specialised treatment to a young person at a later clinical stage (1b or 2–4). (Note that illness progression, disengagement, and recovery are not weighted by clinical stage in our analyses, so that recovery of a patient at stage 1a, for example, is assumed to be equivalent to recovery of a patient at stage 1b or stage 2. Although this may appear problematic, given the higher severity of symptoms and functional impairment at later clinical stages, it should be emphasised that recovery at earlier stages may often prevent more severe symptoms and impairment developing in the first place. This issue is considered further in the Limitations section of the Discussion and in S2 Appendix.)

**Fig 4:**
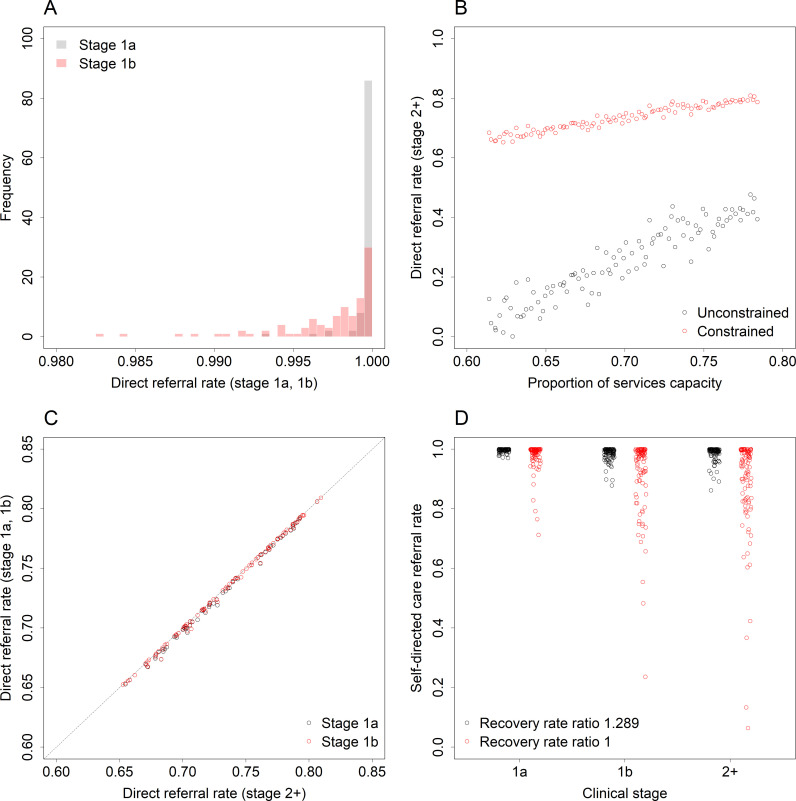
Optimal rates of direct engagement with specialised services (panels A–C) and self-directed care (panel D). Panel A—optimal rates of direct referral to specialised care for patients at stage 1a and stage 1b (i.e., the proportions of patients referred directly to specialised services that minimise total illness progression and disengagement per year). Panel **B—**optimal rates of direct referral to specialised care for patients at stage 2+ as a function of specialised services capacity (expressed as a proportion of total services capacity). In the unconstrained case (black points), no restrictions were placed on the direct referral rates for each clinical stage (except that they had to lie between 0 and 1); in the constrained case (red points), the direct referral rate for patients at stage 1b was restricted to be less than or equal to that for patients at stage 2+ and the direct referral rate for patients at stage 1a was restricted to be less than or equal to that for patients at stage 1b. Panel **C—**optimal rates of direct referral to specialised care for the constrained case. Panel **D—**optimal rates of direct referral to self-directed care (e-health interventions); these rates apply to patients not referred directly to specialised services and correspond to the proportions of patients referred directly to self-directed (online) care that minimise total disease progression and disengagement per year (direct referral rates for online and specialised care were optimised simultaneously, constraining specialised care referral rates for earlier clinical stages to be less than or equal to rates for later clinical stages).

**Fig 5:**
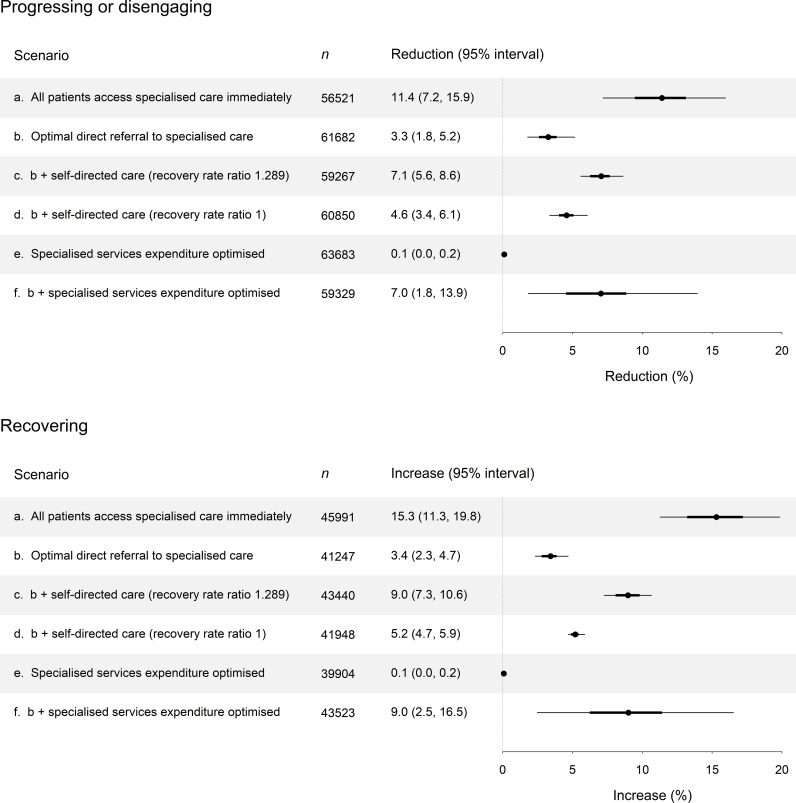
Effects of services system interventions on total numbers of patients progressing or disengaging per year (upper panel) and recovering per year (lower panel). Reductions in progression and disengagement and increases in recovery (rightmost columns and the plots) were calculated relative to a reference scenario in which patients are referred to specialised services only via primary care services (in this scenario, 63752 patients progress or disengage per year and 39876 patients recover per year). Scenario a, in which all patients receive immediate specialised care (no waiting), provides an indication of the maximum possible improvement in outcomes given the assumed parameter values (stage-specific recovery rates, progression rates, disengagement rate, etc.). Scenario b corresponds to the constrained analyses in Fig 4.

Total illness progression and disengagement per year are minimised when young people at earlier clinical stages are preferentially referred directly to specialised services (i.e., when young people at stages 1a and 1b are referred directly to specialised services at a higher rate than young people at stages 2–4; see panels A and B in Fig 4). This result follows immediately from the results presented in Fig 3 but leads to the ethically unacceptable policy recommendation that young people with relatively mild illness should receive specialised treatment before young people experiencing more severe symptoms and functional impairment. Presuming we are not prepared to preferentially refer adolescents and young adults at earlier clinical stages (1a and 1b) directly to specialised care, illness progression and disengagement are minimised when all young people engaging with the mental health care system are referred directly to specialised services at the same rate (irrespective of clinical stage; see Fig 4, panel C). The optimal rate of direct referral to specialised care increases as specialised services capacity increases (Fig 4, panel B), approaching a value of 1 (i.e., all young people presenting for care are referred directly to specialised services) as the proportion of total mental health care expenditure (and therefore total services capacity) allocated to specialised services approaches 1. Assuming the proportion of total expenditure allocated to specialised services is less than 1 (so that the optimal direct referral rate is also less than 1), further reductions in illness progression and disengagement are possible when young people not referred directly to specialised care are referred directly to self-directed online care (see Fig 5; Fig 4, panel D).

## Discussion

The results presented above indicate that policy decisions aimed at optimising services system composition (i.e., the division of total services capacity between primary care services and specialised services), initial access to specialised treatment, and the use of e-health interventions in routine clinical practice have the potential to significantly improve system-level (aggregate) outcomes for adolescents and young adults engaging with mental health services (see Fig 5). Progression to later clinical stages and disengagement from care are minimised where young people are not limited to accessing specialised services only via primary care services and all young people engaging with the health care system are referred directly to specialised services at the same rate, irrespective of clinical stage (see Fig 4, panel C). Adolescents and young adults at earlier clinical stages recover more rapidly when treated than those experiencing more severe symptoms and functional impairment and are more likely to progress to later clinical stages, so that restricting the ability of young people with relatively mild mental health problems to access specialised care (an explicit objective of stepped care models) constrains the capacity of specialised services to promote recovery and prevent illness progression; better system-level outcomes are achieved when young people receive more effective (i.e., specialised) treatment as early as possible. Assuming young people are optimally referred directly to specialised services, total disengagement and illness progression per year decline as the proportion of total mental health care expenditure allocated to specialised services increases (at least for the range of parameter values considered here; Table 1), while the rate of direct referral to specialised care approaches a value of 1 (i.e., direct referral of all young people seeking help), suggesting that the largest improvements in services system performance are likely to be achieved by expanding the capacity of specialised services with the aim of referring all young people presenting for care directly to these services.

Although our simulation analyses suggest that increasing the accessibility of specialised services (as opposed to general practitioner and other non-specialised services) should be prioritised in mental health services planning (at least for young people; see Fig 2, panel B), policies aimed at expanding currently limited numbers of specialised mental health care providers (i.e., psychiatrists, clinical psychologists, etc.) would not be expected to increase available capacity immediately (due to training lead times) [29]. Accordingly, we may reasonably assume that primary care services will continue to account for a significant proportion of total mental health services capacity in the near to medium term, so that the optimal rate of direct referral to specialised care will generally be less than the maximum value of 1 (Fig 4). The results presented in Fig 5 (see also Fig 4, panel D) indicate that where services capacity constraints restrict the accessibility of specialised care (i.e., where the optimal direct referral rate is less than 1), providing young people unable to access timely specialised treatment with immediate access to evidence-based e-health interventions (self-directed online care) has the potential to reduce total disengagement and disease progression per year and increase total recovery per year, even when these interventions are assumed to be no more effective than primary care (in this case, improved health care outcomes result from reduced waiting times). Catarino et al. [30] recently presented an economic evaluation of treatments for mood and anxiety disorders offered as part of the National Health Service’s Talking Therapies program (United Kingdom), providing evidence that online cognitive behavioural therapy (CBT) delivers improved patient outcomes (greater health-related quality of life) and lower health system costs when compared with standard therapy. Deterministic sensitivity analyses indicated that the health and economic benefits of online CBT are attributable primarily to reduced waiting and treatment times, consistent with our general conclusion that facilitating rapid access to effective care, including through the use of evidence-based e-health interventions, is essential for optimising the performance of mental health services systems.

### Limitations

There are at least four principal limitations of the analyses presented here that should be pointed out. Firstly, our dynamic model effectively disregards potentially significant heterogeneity in individual-level factors that are expected to affect rates of disengagement, progression to more severe illness, and treatment-dependent recovery (including principal diagnosis, comorbidities, gender, participation in education or employment) [22,31], and no distinction is made between different types of primary care services (general practitioner services, youth-specific services) and specialised services (psychiatry, clinical psychology, multidisciplinary mental health care, etc.). Analyses of more complex models [32] are needed to determine the extent to which these significant simplifying assumptions (i.e., that young people at the same clinical stage and different types of primary care services and specialised services can be treated as essentially identical) may influence our conclusions. Secondly, for simplicity, we have assumed that access to mental health treatment does not alter the proportions of adolescents and young adults at each clinical stage at the time of initial engagement with the health care system (these proportions are assumed to be constant; see Table 1). Nevertheless, at least in principle, increases in treatment-mediated recovery resulting from improved services system functioning (i.e., following changes in the allocation of expenditure and access to specialised care and online services) have the potential to reduce the proportion of young people presenting for care with full-threshold mental disorders (via a decrease in prevalence) [10,33], increasing the capacity of services to deliver further improvements in system-level outcomes [34]. Thirdly, our model parameter estimates are derived predominantly from data for Australia (see Table 1), so that the applicability of our results in other contexts, and especially in low- and middle-income countries, may be limited. Our conclusions about optimal services system composition, in particular, are not expected to hold where the cost ratio for specialised care (i.e., the cost per patient per year for specialised care divided by the corresponding cost for primary care) is substantially higher than the value of 1.44 assumed in our modelling (see S3 Appendix).

The fourth limitation relates to the measures of services system performance employed in our analyses (i.e., total illness progression and disengagement per year and total recovery per year). As noted in the Results section above, illness progression, disengagement, and recovery are not weighted by clinical stage in calculating these measures, so that recovery of a patient at stage 1a, for example, is assumed to be equivalent to recovery of a patient at stage 1b or stage 2. Although recovery at earlier clinical stages may prevent more severe symptoms and functional impairment from ever developing (which is clearly preferrable to recovery after symptom severity and functional impairment have increased), not all young people will progress to later stages without treatment, while those at later clinical stages have a substantially increased risk of experiencing acute psychiatric symptoms requiring urgent care (suicidality, psychosis, mania, etc.). Depending on how particular system-level goals (e.g., maximising the number of people benefitting from care, minimising functional impairment, preventing suicidal behaviour) are prioritised, there may be compelling reasons for placing greater weight on recovery at later clinical stages in assessing services system performance, which could in turn affect optimal rates of direct referral to specialised services. S2 Appendix presents the results of analyses in which we optimised rates of direct referral to specialised care based on weighted numbers of patients recovering per year, where the weights reflect the relative risks of high levels of psychological distress, significant functional impairment, and suicidal behaviour at each clinical stage [35,36]. Optimal direct referral rates vary considerably depending on the weighting scheme applied, indicating that a clearly specified measure of services system performance (and therefore a clear idea of which outcomes should be prioritised) is critical for decision making aimed at maximising the effectiveness and efficiency of youth mental health care delivery.

## Conclusion

Policy interventions designed to promote early access to effective care are critical both for directly reducing the substantial (current) disability attributable to youth mental disorders, and for preventing persistent, adverse socioeconomic and health outcomes associated with more severe and/or prolonged psychological symptoms and functional impairment [10]. The dynamic modelling analyses presented here indicate that services delivery reforms enabling adolescents and young adults to engage directly with specialised treatment, irrespective of clinical stage, have the potential to significantly improve services system performance, reducing total numbers of young people disengaging from care or progressing to more severe illness per year and increasing the total recovery rate (at equilibrium). Provided that young people are not restricted to accessing specialised care only via primary care services, disengagement from treatment and progression to later clinical stages decline as the proportion of mental health care expenditure allocated to specialised services increases, while additional improvements in system-level outcomes may be achieved by providing young people unable to access timely specialised treatment (due to limited services capacity) with immediate access to evidence-based e-health interventions. Although our results provide no support for differentially referring patients directly to specialised care according to clinical stage (at least where individual-level outcomes are weighted equally; see S2 Appendix), it should be emphasised that a clinical staging perspective is central to the conceptual framework on which these results are based and remains invaluable for clinical decision making (e.g., in selecting specific treatments) [37]; there is no implication here that the utility of clinical stage models for understanding and treating youth mental disorders is in any way diminished.

## Supporting information

S1 Appendix Dynamic model details. (DOCX)

S2 Appendix Optimisation analyses using weighted measures of services system performance. (DOCX)

S3 Appendix Optimal expenditure on specialised services for varying cost ratios. (DOCX)
