# Supplementary material for: Reshaping youth mental health care for optimal system-level outcomes: A dynamic modelling analysis
Source: PLOS Ment Health. 2025 Feb 24;2(2):e0000232. doi: 10.1371/journal.pmen.0000232 (PMC12798356; doi:10.1371/journal.pmen.0000232)
Supplement: S1 Appendix — (DOCX) [file pmen.0000232.s001.docx]

S1 Appendix

Dynamic model details

The dynamic model developed for the analyses comprises three interconnected components, capturing access to primary care services, specialised services, and self-directed online care (i.e., e-health interventions). Figs S1 and S2 show the structure of the primary care services and self-directed care components of the model; the structure of the specialised services component is presented in Fig. 1 of the paper. Denoting the numbers of young people at clinical stage $i$ currently waiting for and receiving primary care as $W_{i}^{p}$ and $T_{i}^{p}$, respectively, we assume that:

$$\frac{dW_{0}^{p}}{dt}=\left[ 1-\theta_{0}-\phi_{0}\left( 1-\theta_{0} \right) \right]q_{0}N-\frac{W_{0}^{p}}{\sum_{i} W_{i}^{p}}\left( \frac{C^{p}-\sum_{i} T_{i}^{p}}{d} \right)-uW_{0}^{p}-\left( h+a+m_{0} \right)W_{0}^{p}$$

$$\frac{dT_{0}^{p}}{dt}=\frac{W_{0}^{p}}{\sum_{i} W_{i}^{p}}\left( \frac{C^{p}-\sum_{i} T_{i}^{p}}{d} \right)-uT_{0}^{p}-\left( r_{0}+f_{0}+h+a+m_{0} \right)T_{0}^{p}$$

$$\frac{dW_{1}^{p}}{dt}=\left[ 1-\theta_{1}-\phi_{1}\left( 1-\theta_{1} \right) \right]q_{1}N-\frac{W_{1}^{p}}{\sum_{i} W_{i}^{p}}\left( \frac{C^{p}-\sum_{i} T_{i}^{p}}{d} \right)-vW_{1}^{p}+uW_{0}^{p}-\left( h+a+m_{1} \right)W_{1}^{p}$$

$$\frac{dT_{1}^{p}}{dt}=\frac{W_{1}^{p}}{\sum_{i} W_{i}^{p}}\left( \frac{C^{p}-\sum_{i} T_{i}^{p}}{d} \right)-vT_{1}^{p}+uT_{0}^{p}-\left( r_{1}+f_{1}+h+a+m_{1} \right)T_{1}^{p}$$

$$\frac{dW_{2}^{p}}{dt}=\left[ 1-\theta_{2}-\phi_{2}\left( 1-\theta_{2} \right) \right]q_{2}N-\frac{W_{2}^{p}}{\sum_{i} W_{i}^{p}}\left( \frac{C^{p}-\sum_{i} T_{i}^{p}}{d} \right)+vW_{1}^{p}-\left( h+a+m_{2} \right)W_{2}^{p}$$

$$\frac{dT_{2}^{p}}{dt}=\frac{W_{2}^{p}}{\sum_{i} W_{i}^{p}}\left( \frac{C^{p}-\sum_{i} T_{i}^{p}}{d} \right)+vT_{1}^{p}-\left( r_{2}+f_{2}+h+a+m_{2} \right)T_{2}^{p}\text{,}$$

where $i$ is equal to 0 for clinical stage 1a, 1 for stage 1b, and 2 for stages 2−4, and all other notation is as defined in Table S1. In words, stage-specific rates of change in the numbers of young people waiting for primary care, ${{dW}_{i}^{p}}/{dt}$, are equal to the numbers of young people at each clinical stage engaging with primary care services per year, given by $\left[ 1-{\theta_{i}-\phi}_{i}\left( 1-\theta_{i} \right) \right]q_{i}N$, minus the numbers of young people at each stage starting primary care per year, equal to $\left( {W_{i}^{p}}/{\sum_{i} W_{i}^{p}} \right)/\left[ \left( C^{p}-\sum_{i} T_{i}^{p} \right)/d \right]$, plus any changes in the stage-specific numbers of young people waiting for primary care associated with disease progression ($-uW_{0}^{p}$ for stage 1a, $-vW_{1}^{p}+uW_{0}^{p}$ for stage 1b, and $vW_{1}^{p}$ for stages 2−4), minus the numbers of young people waiting for primary care leaving the


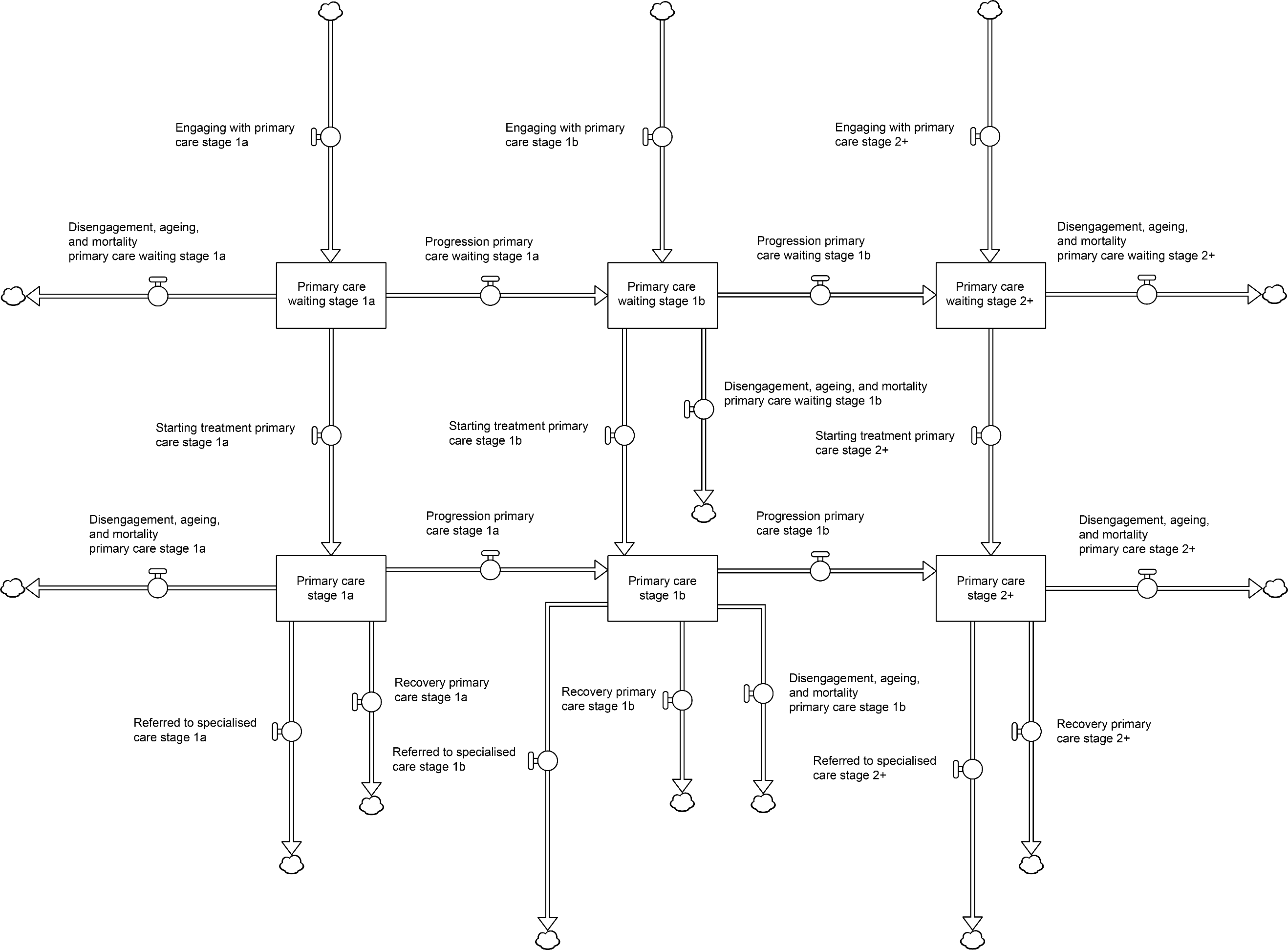


Fig. S1. Structure of the primary mental health care section of the model. Stocks (or compartments) are shown as boxes, flows as pipes with taps, and sources and sinks as clouds (see Homer and Hirsch, 2006). Figs 1 and S2 present the structure of the specialised services and self-directed care (e-health interventions) components of the model.


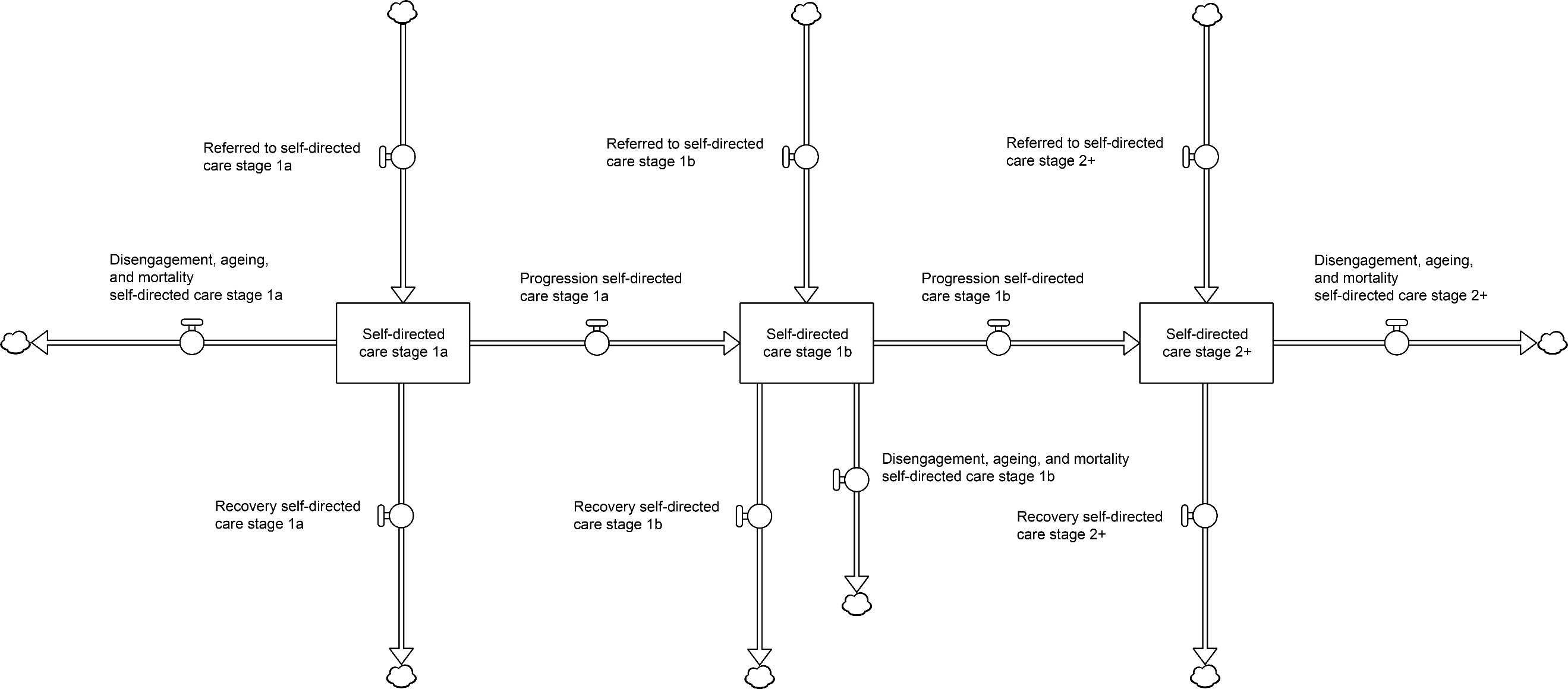


Fig. S2. Structure of the self-directed care component of the model.

services system per year due to disengagement, ageing, and mortality, equal to $\left( h+a+m_{i} \right)W_{i}^{p}$. Stage-specific rates of change in the numbers of young people receiving primary care, ${{dT}_{i}^{p}}/{dt}$, are equal to the numbers of young people at each stage starting primary care per year, plus any changes in the stage-specific numbers of young people receiving primary care associated with illness progression ($-uT_{0}^{p}$ for stage 1a, $-vT_{1}^{p}+uT_{0}^{p}$ for stage 1b, and $vT_{1}^{p}$ for stages 2−4), minus the stage-specific numbers of young people leaving primary care services per year due to recovery, referral to specialised services, disengagement, ageing, and mortality (the last term in each equation).

Letting $W_{i}^{s}$ and $T_{i}^{s}$ denote the numbers of young people at clinical stage $i$ currently waiting for and receiving specialised care, the dynamics of the specialised services component of the model are given by:

$$\frac{dW_{0}^{s}}{dt}=\theta_{0}q_{0}N+f_{0}T_{0}^{p}-\frac{W_{0}^{s}}{\sum_{i} W_{i}^{s}}\left( \frac{C^{s}-\sum_{i} T_{i}^{s}}{d} \right)-uW_{0}^{s}-\left( h+a+m_{0} \right)W_{0}^{s}$$

$$\frac{dT_{0}^{s}}{dt}=\frac{W_{0}^{s}}{\sum_{i} W_{i}^{s}}\left( \frac{C^{s}-\sum_{i} T_{i}^{s}}{d} \right)-uT_{0}^{s}-\left( {\gamma r}_{0}+h+a+m_{0} \right)T_{0}^{s}$$

$$\frac{dW_{1}^{s}}{dt}=\theta_{1}q_{1}N+f_{1}T_{1}^{p}-\frac{W_{1}^{s}}{\sum_{i} W_{i}^{s}}\left( \frac{C^{p}-\sum_{i} T_{i}^{s}}{d} \right)-vW_{1}^{s}+uW_{0}^{s}-\left( h+a+m_{1} \right)W_{1}^{s}$$

$$\frac{dT_{1}^{s}}{dt}=\frac{W_{1}^{s}}{\sum_{i} W_{i}^{s}}\left( \frac{C^{s}-\sum_{i} T_{i}^{s}}{d} \right)-vT_{1}^{s}+uT_{0}^{s}-\left( \gamma r_{1}+h+a+m_{1} \right)T_{1}^{s}$$

$$\frac{dW_{2}^{s}}{dt}=\theta_{2}q_{2}N+f_{2}T_{2}^{p}-\frac{W_{2}^{s}}{\sum_{i} W_{i}^{s}}\left( \frac{C^{s}-\sum_{i} T_{i}^{s}}{d} \right)+vW_{1}^{s}-\left( h+a+m_{2} \right)W_{2}^{s}$$

$$\frac{dT_{2}^{s}}{dt}=\frac{W_{2}^{s}}{\sum_{i} W_{i}^{s}}\left( \frac{C^{s}-\sum_{i} T_{i}^{s}}{d} \right)+vT_{1}^{s}-\left( {\gamma r}_{2}+h+a+m_{2} \right)T_{2}^{s}\text{,}$$

where all notation is as defined above and in Table S1. These equations are interpreted in the same way as the equations for primary care services. Stage-specific numbers of young people waiting for specialised care change over time due to direct engagement with specialised services ($\theta_{i}q_{i}N$ young people per year), treatment initiation, illness progression, and disengagement, ageing, and mortality, as well as through referrals from primary care services ($f_{i}T_{i}^{p}$ young people per year). Rates of change in stage-specific numbers of young people receiving specialised care similarly depend on treatment initiation, illness progression, and recovery, disengagement, ageing, and mortality. The equations describing the dynamics of the self-directed online care component of the model are:

$$\frac{dT_{0}^{o}}{dt}=\phi_{0}\left( 1-\theta_{0} \right)q_{0}N-uT_{0}^{o}-\left( {\eta r}_{0}+h+a+m_{0} \right)T_{0}^{o}$$

$$\frac{dT_{1}^{o}}{dt}=\phi_{1}\left( 1-\theta_{1} \right)q_{1}N-vT_{1}^{o}+uT_{0}^{o}-\left( \eta r_{1}+h+a+m_{1} \right)T_{1}^{o}$$

$$\frac{dT_{2}^{o}}{dt}=\phi_{2}\left( 1-\theta_{2} \right)q_{2}N+vT_{1}^{o}-\left( {\eta r}_{2}+h+a+m_{2} \right)T_{2}^{o}\text{,}$$

where $T_{0}^{o}$, $T_{1}^{o}$, and $T_{2}^{o}$ are, respectively, the numbers of young people at clinical stages 1a, 1b, and 2−4 currently receiving self-directed e-health interventions, and all other notation is as defined above and in Table S1. Note that there are no waiting stocks for self-directed care (see Fig S2), since services capacity is effectively unconstrained; young people flow directly into the treatment stocks at rates (per year) equal to $\phi_{i}\left( 1-\theta_{i} \right)q_{i}N$.

References

Australian Bureau of Statistics, 2020. Deaths, Australia. Cat. no. 3302.0. Australian Bureau of Statistics, Canberra.

Carpenter, J. S., Iorfino, F., Cross, S. P., Davenport, T. A., Hermens, D. F., Rohleder, C., Crouse, J. J., Leweke, F. M., Koethe, D., Guastella, A. J., Naismith, S. L., Scott, J., Scott, E. M., Hickie, I. B., 2019. Combining clinical stage and pathophysiological mechanisms to understand illness trajectories in young people with emerging mood and psychotic syndromes. Med. J. Aust. 211 (9 Suppl.), S12−S22.

Cuijpers, P., van Straten, A., van Schaik A., Andersson, G., 2009. Psychological treatment of depression in primary care: a meta-analysis. Br. J. Gen. Pract., doi: 10.3399/bjgp09X395139.

Homer, J. B., Hirsch, G. B., 2006. System dynamics modeling for public health: background and opportunities. Am. J. Public Health 96, 452−458.

Iorfino, F., Scott, E. M., Carpenter, J. S., Cross, S. P., Hermens, D. F., Killedar, M., Nichles, A., Zmicerevska, N., White, D., Guastella, A. J., Scott, J., McGorry, P. D., Hickie, I. B., 2019. Clinical stage transitions in persons aged 12 to 25 years presenting to early intervention mental health services with anxiety, mood, and psychotic disorders. J. Am. Med. Assoc. Psychiatry 76, 1167−1175.

KPMG, 2022. Evaluation of the national headspace program. Final report, Department of Health, June 2022. Available at: https://www.health.gov.au/sites/default/files/documents/2022/10/evaluation-of-the-national-headspace-program.pdf.

Massoudi, B., Holvast, F., Bockting, C. L. H., Burger, H., Blanker, M. H., 2019. The effectiveness and cost-effectiveness of e-health interventions for depression and anxiety in primary care: a systematic review and meta-analysis. J. Affect. Disord. 245, 728−743.

Mei, C., McGorry, P. D., Hickie, I. B., 2019. Clinical staging and its potential to enhance mental health care. In: McGorry, P. D., Hickie, I. B. (Eds.), Clinical staging in psychiatry. Cambridge University Press, Cambridge, pp. 12−33.

Mulraney, M., Lee, C., Freed, G., Sawyer, M., Coghill, D., Sciberras, E., Efron, D., Hiscock, H., 2021. How long and how much? Wait times and costs for initial private child mental health appointments. J. Paediatr. Child Health 57, 526−532.

Tyrer, P., Morgan, J., Van Horn, E., Jayakody, M., Evans, K., Brummell, R., White, T., Baldwin, D., Harrison-Read, P., Johnson, T., 1995. A randomised controlled study of close monitoring of vulnerable psychiatric patients. Lancet 345, 756−759.

Walker, E. R., McGee, R. E., Druss, B. G., 2015. Mortality in mental disorders and global disease burden implications: a systematic review and meta-analysis. J. Am. Med. Assoc. Psychiatry 72, 334−341.

Table S1. Model parameters. Distributions are given for the values of parameters included in the sensitivity analyses (see Methods section of the paper).

| Parameter | Symbol | Value(s) | Reference(s) |
| --- | --- | --- | --- |
|  |  |  |  |
| Mental health care engagement rate per year | *N* | 100000 |  |
| Proportions of young people engaging with care at each clinical stage | *q_i_* | 0.467 (stage 1a); 0.238 (stage 1b); 0.296 (stage 2+) | Mei et al. (2019) |
| Proportions of young people engaging directly with specialised services | *θ_i_* | See Fig. 4 of the paper |  |
| Proportions of young people not referred directly to specialised services engaging directly with self-directed online care | *φ_i_* | See Fig. 4 of the paper |  |
| Per capita rate of progression from stage 1a to stage 1b per year | *u* | Mean 0.253, 95% equal-tail interval 0.213−0.294 (lognormal distribution) | Iorfino et al. (2019) |
| Per capita rate of progression from stage 1b to stage 2+ per year | *v* | Mean 0.060, 95% equal-tail interval 0.047−0.073 (lognormal distribution) | Iorfino et al. (2019) |
| Per capita disengagement rate per year | *h* | 0.262 | Tyrer et al. (1995) |
| Proportion of young people reaching age 26 per year | *a* | 0.0714 |  |
| Per capita mortality rate per year (stage-specific) | *m_i_* | 0.000213 (stages 1a and 1b), 0.00047286 (stage 2+) | Australian Bureau of Statistics (2020); Walker et al. (2015) |
| Primary care services capacity | *C^p^* | 34674−68938 | Australian Institute of Health and Welfare (data available at: https://www.aihw.gov.au/mental-health/resources/data-tables) |
| Specialised services capacity | *C^s^* | 102245−140100 | Australian Institute of Health and Welfare (data available at: https://www.aihw.gov.au/mental-health/resources/data-tables) |
| Treatment initiation delay (determines how rapidly new patients start treatment as services capacity becomes available) | *d* | 0.0192 (1 week) |  |
| Rate of referral from primary care services to specialised services per year (stage-specific) | *f_i_* | 0.441−1.956 (stage 1a), 0.836−2.855 (stage 1b), 1.991−4.648 (stage 2+) | Mulraney et al. (2021); KPMG (2022) |
| Per capita recovery rate per year, primary care (stage-specific) | *r_i_* | 0.259 (stage 1a); 0.129 (stage 1b); 0.091 (stage 2+) | Carpenter et al. (2019) |
| Recovery rate ratio, specialised care | *γ* | Mean 1.921, 95% equal-tail interval 1.490−2.477 (lognormal distribution) | Cuijpers et al. (2009) |
| Recovery rate ratio, self-directed care (e-health interventions) | *η* | 1.289 | Massoudi et al. (2019) |
|  |  |  |  |
