# Supplementary material for: Reshaping youth mental health care for optimal system-level outcomes: A dynamic modelling analysis
Source: PLOS Ment Health. 2025 Feb 24;2(2):e0000232. doi: 10.1371/journal.pmen.0000232 (PMC12798356; doi:10.1371/journal.pmen.0000232)
Supplement: S2 Appendix — (DOCX) [file pmen.0000232.s002.docx]

S2 Appendix

Results of optimisation analyses using weighted measures of services system performance

Fig. S3 presents the results of analyses in which we optimised rates of direct referral to specialised care based on weighted numbers of patients recovering per year, where the weights reflect the relative risks of high to very high psychological distress (K10 score > 21), significant functional (psychosocial) impairment (SOFAS score ≤ 60), and suicidal behaviour (suicide attempt in the past year) at each clinical stage (Table S2). Optimal stage-specific rates of direct referral to specialised services under the psychological distress (K10) weighting scheme are nearly identical to those for the equal weighting scheme used in our primary analyses (i.e., the analyses presented in the paper); however, optimal referral rates under the functional impairment (SOFAS) and suicidal behaviour (suicide) weighting schemes differ substantially from those for the equal and psychological distress weighting schemes. More young people at stages 2−4 are referred directly to specialised care under the functional impairment and suicidal behaviour weighting schemes (due to significantly higher risks of functional impairment and suicidal behaviour), resulting in higher numbers of stage 2−4 patients recovering per year (see Fig. S3, upper right panel). Nevertheless, substantially lower direct referral rates for patients at stage 1a (functional impairment and suicidal behaviour weighting schemes) and stage 1b (impairment weighting scheme) mean that total numbers of patients recovering per year and progressing or disengaging per year are lower and higher, respectively (Fig. S3, lower panels).

Table S2. Alternative weighting schemes for recovery at each clinical stage used in evaluating services system performance (see Fig. S3). All weights are relative to recovery of a patient at stage 1a; e.g., recovery of a patient at stage 1b is equivalent to recovery of 1.167 stage 1a patients under the psychological distress weighting scheme, 1.814 stage 1a patients under the functional impairment weighting scheme, and 6.962 stage 1a patients under the suicidal behaviour weighting scheme.

| Weighting scheme | Stage 1a | Stage 1b | Stages 2−4 | Reference |
| --- | --- | --- | --- | --- |
|  |  |  |  |  |
| Equal | 1 | 1 | 1 |  |
| High-very high psychological distress (K10 score > 21) | 1 | 1.167 | 1.346 | Scott et al. (2012) |
| Functional impairment (SOFAS score ≤ 60) | 1 | 1.814 | 2.903 | Scott et al. (2012) |
| Suicidal behaviour (suicide attempt in past year) | 1 | 6.962 | 12.654 | Capon et al. (2022) |
|  |  |  |  |  |

References

Capon, W., Hickie, I. B., Varidel, M., Prodan, A., Crouse, J. J., Carpenter, J. S., Cross, S. P., Nichles, A., Zmicerevska, N., Guastella, A. J., Scott, E. M., Scott, J., Shah, J., Iorfino, F., 2022. Clinical staging and the differential risks for clinical and functional outcomes in young people presenting for youth mental health care. BMC Med. 20, 479.

Scott, E. M., Hermens, D. F., Glozier, N., Naismith, S. L., Guastella, A. J., Hickie, I. B., 2012. Targeted primary care-based mental health services for young Australians. Med. J. Aust. 196, 136−140.


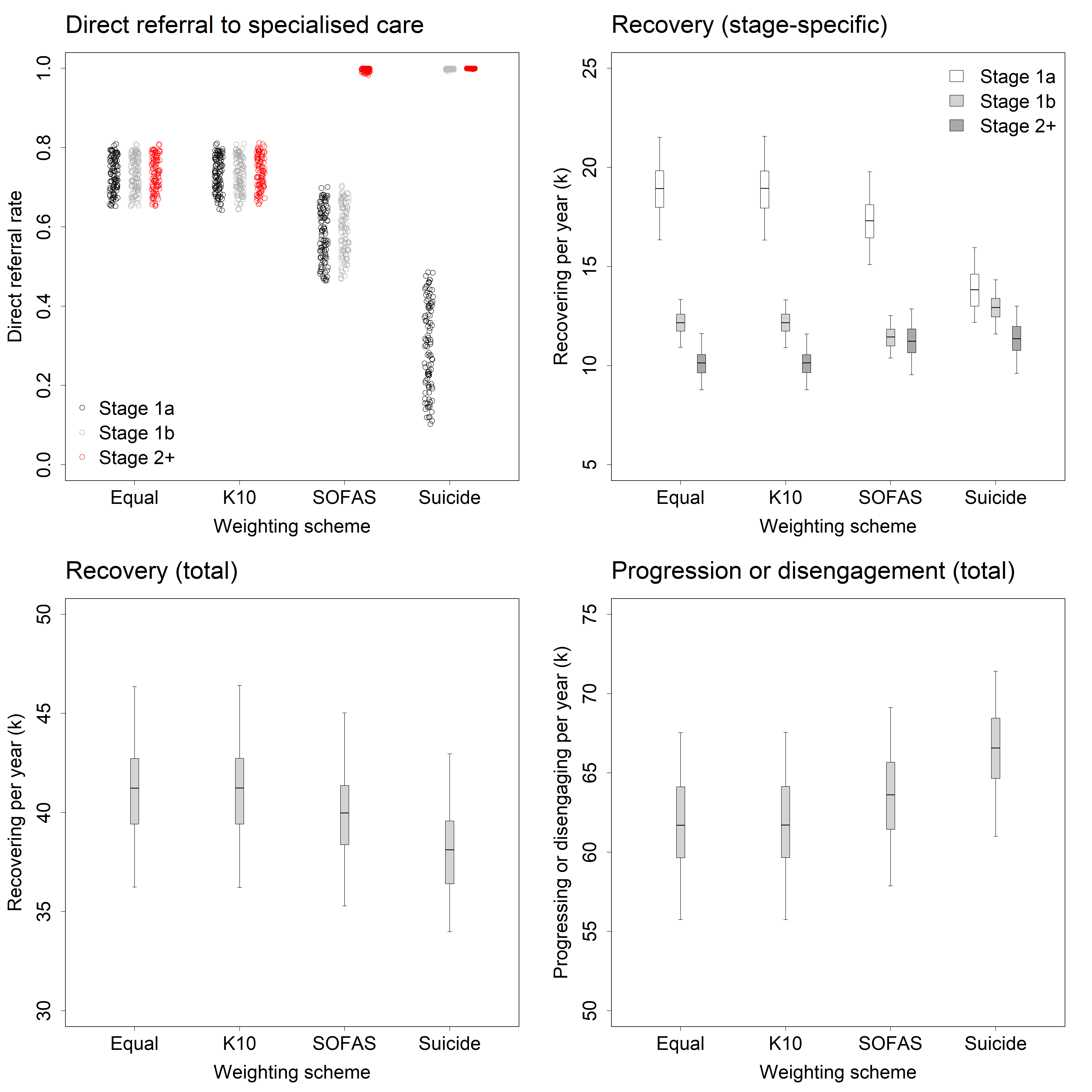


Fig. S3. Optimal stage-specific rates of direct referral to specialised care, stage-specific and total numbers of young people recovering per year, and total numbers of young people disengaging or progressing to more severe illness per year for analyses using weighted measures of services system performance (see Table S2). Mean values (horizontal bars), 50% and 95% intervals (boxes and error bars, respectively), and individual points are derived from sensitivity analyses (see Methods section and Table 1 of the paper).
