# Supplementary material for: Reshaping youth mental health care for optimal system-level outcomes: A dynamic modelling analysis
Source: PLOS Ment Health. 2025 Feb 24;2(2):e0000232. doi: 10.1371/journal.pmen.0000232 (PMC12798356; doi:10.1371/journal.pmen.0000232)
Supplement: S3 Appendix — (DOCX) [file pmen.0000232.s003.docx]

S3 Appendix

Optimal expenditure on specialised services for varying specialised services cost ratios


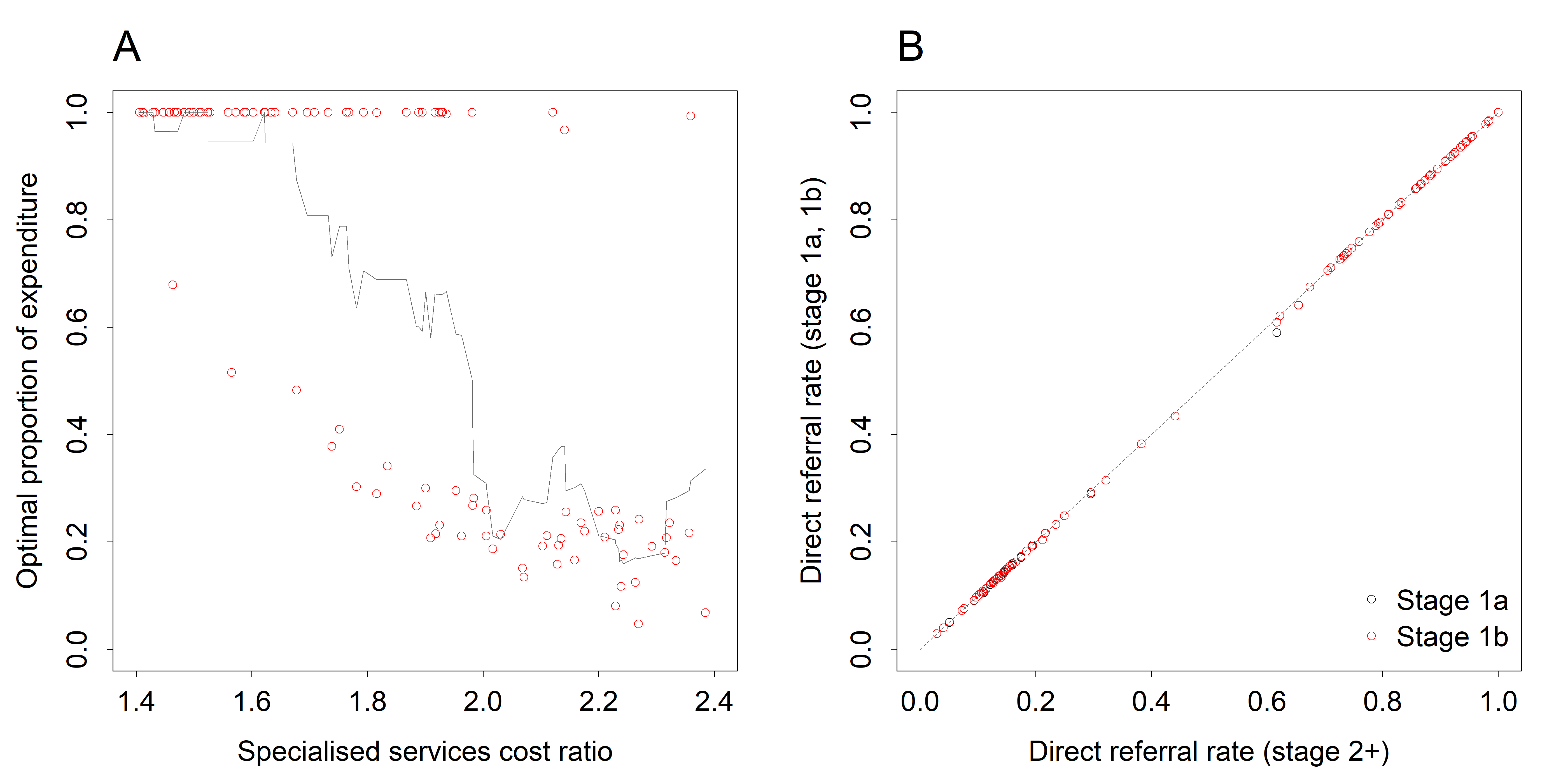


Fig. S4. Panel A — effect of the cost ratio for specialised care on the optimal proportion of total mental health care expenditure allocated to specialised services (i.e., the proportion of expenditure allocated to specialised services minimising the total illness progression and disengagement rate). Individual points are derived from a sensitivity analysis in which the specialised services cost ratio was sampled from a uniform distribution (minimum 1.4, maximum 2.4) and the values of all remaining parameters were either optimised (rates of direct referral to specialised care; see panel B) or obtained using the same approach described for the sensitivity analyses in the paper (see Methods section and Table 1); the solid grey line is a simple moving average. Except for the varying specialised services cost ratio, the simulations correspond to scenario f in Fig. 5. Note that for cost ratios above *c*. 2, the optimal proportion of total expenditure allocated to specialised services is substantially less than 1. Panel B — optimal stage-specific rates of direct referral to specialised services for the simulations presented in panel A. Although the optimal allocation of expenditure to specialised services depends on the specialised services cost ratio, our conclusion that all young people should be referred directly to specialised services at the same rate, irrespective of clinical stage, holds generally; for all simulations, the optimal referral rates for each clinical stage are the same (or nearly so), consistent with the results in panel C of Fig. 4.
